# Integrating microbial community properties, biomass and necromass to predict cropland soil organic carbon

**DOI:** 10.1038/s43705-023-00300-1

**Published:** 2023-08-23

**Authors:** Chao Wang, Xu Wang, Yang Zhang, Ember Morrissey, Yue Liu, Lifei Sun, Lingrui Qu, Changpeng Sang, Hong Zhang, Guochen Li, Lili Zhang, Yunting Fang

**Affiliations:** 1 CAS Key Laboratory of Forest Ecology and Management, Institute of Applied Ecology, Chinese Academy of Sciences, Shenyang, 110016 China; 2 Key Laboratory of Terrestrial Ecosystem Carbon Neutrality, Liaoning Province, Shenyang, 110016 China; 3 Division of Plant and Soil Sciences, West Virginia University, Morgantown, 26506 USA

**Keywords:** Biogeochemistry, Microbial ecology

## Abstract

Manipulating microorganisms to increase soil organic carbon (SOC) in croplands remains a challenge. Soil microbes are important drivers of SOC sequestration, especially via their necromass accumulation. However, microbial parameters are rarely used to predict cropland SOC stocks, possibly due to uncertainties regarding the relationships between microbial carbon pools, community properties and SOC. Herein we evaluated the microbial community properties (diversity and network complexity), microbial carbon pools (biomass and necromass carbon) and SOC in 468 cropland soils across northeast China. We found that not only microbial necromass carbon but also microbial community properties (diversity and network complexity) and biomass carbon were correlated with SOC. Microbial biomass carbon and diversity played more important role in predicting SOC for maize, while microbial network complexity was more important for rice. Models to predict SOC performed better when the microbial community and microbial carbon pools were included simultaneously. Taken together our results suggest that microbial carbon pools and community properties influence SOC accumulation in croplands, and management practices that improve these microbial parameters may increase cropland SOC levels.

## Introduction

Given the massive potential (~3.2 Pg C yr^−1^) for carbon sequestration in terrestrial soils [1], there is an imminent need to identify cost-effective strategies for fostering C storage in soils [2]. Relative to natural ecosystems, cropland generally has lower soil organic carbon (SOC) as a result of agricultural management practices and crop harvest [3]. For example, fallowing, cultivation and biomass removal can decrease SOC by reducing C inputs to the soil, increasing decomposition rates, or both [4]. Consequently, cropland may present an opportunity for enhanced SOC storage if it is managed strategically and experiences significant harvest residues inputs [5, 6]. Field studies have indicated that long-term return of crop residues could greatly improve cropland SOC stocks by increasing plant-derived C [7, 8]. On a global scale, it has been estimated that cropland soils can sequester 0.90–1.85 Pg C yr^−1^, which is equivalent to ~10% of the current annual fossil fuel emissions [9]. Thus, cropland C sequestration through effective management is considered to be one of the few approaches that could be implemented on a large scale [10, 11].

Soil microorganisms are believed to regulate SOC decomposition and formation [12–14]. Indeed, the modeling studies and field observations have indicated that microbial-derived necromass C, which includes dead cells, cell parts, cellular debris, and extracellular polymeric substances, can contribute as much as 50–80% of SOC [15–19]. The persistence of microbial necromass is determined by its chemical composition and its interactions with soil minerals [20–23]. In addition, microbial community properties (diversity, community composition) may influence the formation and persistence of microbial necromass, with consequences for SOC sequestration [12, 24, 25]. First, microbial community composition and species interactions regulate microbial death pathways, which can influence the quantity and molecular composition of necromass [24]. For instance, fungal-derived necromass is comprised of complex, C-rich cell wall fragments [26], while bacterial cell wall fragments contain more N [27]. Regarding interactions, bacteriophage infection could accelerate the lysis of select bacterial populations resulting in more accumulation of bacterial cell wall residues compared to fungal residues [24]. Second, microbial necromass available for mineral stabilization may be regulated by the efficiency of microbial biomass production [28, 29]. Recent evidence suggests microbial community with higher carbon use efficiency and fungal abundance accumulate more microbial-derived organic C [21, 30]. Therefore, there is a close interconnection between microbial community properties, living biomass production, and necromass. However, we currently have limited knowledge on the quantitative relationships between these factors on a large scale, and the unresolved challenge remains as to how to effectively integrate them into SOC prediction models.

Cropland soils are facing SOC depletion, especially in northeast China, where the SOC has decreased from 50 to 24 g kg^−1^ after 150 years of cultivation [31, 32]. There is an urgent need for a comprehensive understanding of the microbial-driven processes involved in SOC formation to restore cropland SOC. Additionally, crop types influence long-term SOC dynamics and sequestration potential. Notably, SOC declines are less pronounced in rice paddies than that in adjacent upland maize fields [33–35]. However, the microbial-mediated mechanisms underlying crop-driven differences in SOC losses are unknown, limiting our ability to crop-specific predictions and recommendations for SOC sequestration.

Here, we conducted a survey of 468 cropland soils taken from Liaoning province in northeast China, including maize (*n* = 349) and rice (*n* = 119). The aims of this study are to: (1) investigate the relationships between microbial community properties (diversity and network complexity), microbial carbon pools (living biomass and necromass) and SOC; and (2) develop crop-specific predictive models that leverage microbial parameters. We hypothesize that microbial necromass C can explain a large proportion of the variation in SOC, but its importance will vary between maize and rice soils. Additionally, we hypothesize that the inclusion of microbial parameters (i.e., community properties and C pools) will improve model predictions of SOC.

## Methods

### Site description and soil sampling

This study was conducted in Liaoning province, located in northeast China (Supplementary Fig. S1), which is one of most important grain production provinces in China, with a land area of ~5.15 million hm^2^ under cultivation. The climate is predominantly temperate and semi-humid in southern Liaoning province, and the western region has a continental monsoon climate; the mean growing season precipitation ranges from 450–1200 mm; the mean growing season temperature ranges from 4.6–10.3°C. The soil types in Liaoning province mainly include Aridisols, Alfisols, Inceptisols, and Entisols in accordance with the USDA soil taxonomy [36].

In total, we collected 468 sampling sites, including 349 maize fields and 119 rice fields, from September and October 2019 after crop harvest. The sampled fields have been under cultivation with maize or rice at least 20 years. At each site, five sampling plots (each of 100 m^2^) were established and five soil cores (2.5 cm in diameter) within 0–15 cm were taken from each plot. Then all 25 samples were mixed thoroughly to generate one composite soil sample for each site. Soil samples were immediately sieved through a 2.0-mm mesh, visible living plant material was manually removed. Soil was transported to the laboratory in a box with dry ice. Subsamples from each site were stored at 4°C prior to measuring soil microbial biomass C, dissolved organic C, dissolved total nitrogen (N), available phosphorus, and water content. Another subsample was air-dried for the analysis of pH, SOC, total nitrogen, total phosphorus, fungal necromass carbon, and bacterial necromass carbon. The remaining soil samples were stored at −80°C for subsequent microbial community DNA extraction.

### Soil physiochemical analysis

Soil pH was measured at an air-dried soil to water ratio of 1:2.5 (soil: water) by a pH electrode (Leici, Shanghai, China). The soil water content (SWC) was determined by oven-drying fresh soil. The SOC and total nitrogen content in air-dried soil were measured with an elemental Flash EA analyzer (Thermo Fisher Scientific, USA) after the soil was ground by a ball mill. Soil total phosphorus was measured using a digestion method. Soil available phosphorus was extracted according to the method described by Olsen-P method and measured using an automated discrete analyzer (SmartChem140, AMS, Italy).

### Microbial biomass and necromass C analysis

Soil microbial biomass C was determined by the fumigation extraction method described by Vance et al. [37]. Briefly, 20 g of fresh soil was extracted with 80 mL of 0.5 M K_2_SO_4_ solution. Then, another 20 g of fresh soil was fumigated with ethanol-free chloroform in the dark for 24 h and extracted with 80 mL of 0.5 M K_2_SO_4_ solution. The concentration of total organic C in each extract was analyzed using a TOC analyzer (TOC, Shimadzu, Kyoto, Japan). The conversion factor used to calculate the MBC was 0.45 [38].

Amino sugars are important indices for the contribution of soil microbial necromass to soil organic matter [39]. Amino sugars were determined according to the protocol of Zhang and Amelung [40]. Three amino sugars (glucosamine (GluN), galactosamine (GalN) and muramic acid (MurA)) were used to quantify the microbial necromass carbon accumulation in soil [39]. We used amino sugar-C contents by normalizing their molecular masses as a proxy to calculate the bacterial necromass C and fungal necromass C contents using the following equations from Joergensen [39] and Liang et al. [41]: bacterial necromass C = MurA × 45, fungal necromass C = (GluN/179.17 - 2 × MurA/251.23) × 179.17 × 9. The total necromass C was estimated as the sum of bacterial necromass C and fungal necromass C.

### Amplicon sequencing and data processing

Soil genomic DNA was extracted from 0.5 g freeze-dried soil using a QIAGEN DNA Isolation Kit according to the manufacturer’s instructions. The quantity and quality of extracted DNA were estimated by using a NanoDrop Spectrophotometer (Thermo Scientific, Waltham, MA, USA). We characterized bacterial and fungal communities by amplifying and sequencing the V4-V5 regions of the 16S rRNA gene using the 515 F (5′- GTG CCA GCM GCC GCG GTA A -3′) / 806 R (5′- GGA CTA CHV GGG TWT CTA AT -3′) primers [42] and the ITS1 region of the ITS genes using ITS1 (5′- CTT GGT CAT TTA GAG GAA GTA A -3′) / ITS2 (5′- GCTGC GTT CTT CAT CGA TGC-3′) primers [43]. PCR amplification for the 16 S gene was performed in triplicate under the following conditions: 95°C for 2 min, followed by 25 cycles at 95°C for 30 s, 55°C for 30 s, and 72°C for 30 s and a final extension at 72°C for 5 min. Sequencing libraries were prepared by using an Illumina Nextera kit. Paired-end sequencing (2 × 250) was performed by using an Illumina MiSeq system (Illumina, San Diego, CA, USA). For ITS gene, the PCRs were carried out in a final volume of 50 μL, comprising 100 ng of template DNA, 25 μL of Phusion Hot start flex 2 × Master Mix, and 2.5 μL of 10 μmol L^-1^ each of the forward and reverse primers, made up to the final volume with double distilled water (ddH_2_O). The same volume of ddH_2_O instead of template DNA was added to the above PCR system as a negative control group. The PCR of ITS1 rDNA was implemented under the following procedures: 3 min at 94°C, followed by 25 cycles of 60 s at 95°C, 60 s at 50°C, and 60 s at 72°C, and then a final 7 min extension step at 72°C was executed using a thermal cycler (Bio-Rad, Hercules, CA, United States). High-throughput sequencing was performed at the Institutional Center for Shared Technologies and Facilities at the Institute of Applied Ecology in Shenyang, China.

The raw sequencing data were qualified through screening and the removal of sequences that were shorter than 200-bp, with a quality score below 20 (Q < 20), contained ambiguous bases or did not exactly match the primer sequences and barcode tags. In addition, the cross-sample singletons and doubletons were removed, which were defined as sequences that occurred only once (singletons) or twice (doubletons) among all samples. Then, the high-quality sequences were processed using VSEARCH [44] and QIIME2 [45]. The sequences were clustered into amplicon sequence variants (ASVs) at a similarity level of 100% by using the UPARSE. Data were rarified to 16450 ASVs for bacteria and 6750 ASVs for fungi across all samples. The Ribosomal Database Project (RDP) classifier tool was used to classify all sequences into different taxonomic groups based on the SILVA (version 138) database [46] for bacterial 16 S rRNA and the UNITE (version 9.0) database [47] for fungal ITS. For assessing microbial diversity, we calculated the alpha-diversity metrics, including the observed species and Shannon index. Microbial beta-diversity was estimated using the Bray-Curtis dissimilarity metric between samples. Beta-diversity of bacterial and fungal communities was quantified using a principal coordinate analysis (PCoA) of Bray-Curtis dissimilarities, visualized on a two-dimensional plot.

### Network construction

Network analysis has proven helpful in deciphering complex microbial interaction patterns [48, 49]. Thus, bacteria-fungi internetworks were constructed for each crop and the network’s topological parameters were extracted to describe the microbial community complexity [49]. Microbial networks were constructed using the “igraph [50]” and “psych [51]” packages based on the Spearman correlation matrix for maize and rice, respectively. Microbial phylotypes with relative abundances less than 0.01% of the total number of bacterial and fungal sequences were excluded from the analysis. Then, the bacterial and fungal ASVs were merged into an abundance table. Pairwise Spearman correlations between ASVs were calculated, and *P*-values were adjusted by the Benjamini and Hochberg false discovery rate (FDR) test. The cutoff of the FDR-adjusted *P*-values was 0.001, and correlations with a coefficient of less than 0.7 were also removed. These criteria allowed us to concentrate on the ASVs that exhibited strong co-occurrence patterns and were more likely to interact with each other.

The network topological parameters of each sample were extracted using the subgraph function in the “igraph” package following Ma et al. [48]. The network topological parameters used in this study included the node number (*n*), average connectivity (average K), centralization of betweenness (CB), clustering coefficient (CC), centralization of degree (CD), density (Den) and average path length (average L). Because these topological parameters were tightly correlated, we used the first and second components (network PC1 and network PC2) of the seven selected topological parameters to denote the network complexity. Finally, the interactive platform “Gephi” was used to identify the modules of microbial taxa that strongly interacted with each other.

### Statistical analyses

We tested the differences in SOC, microbial biomass C, microbial necromass C, soil pH, available phosphorus, total nitrogen to phosphorus (N/P ratio), soil water content and microbial alpha diversity between maize and rice soil using one-way ANOVA. Homogeneity of variances was tested by Levene’s test, and the normal distribution of residues was tested by the Shapiro test. Statistical differences in the microbial community composition were tested using permutational multivariate analysis of variance (PERMANOVA) by the “vegan” package [52]. We used the Wilcoxon rank-sum test to determine the difference in network topological parameters between maize and rice.

### Identifying the best set of predictors for soil organic carbon

Predictors including climate (growth season precipitation and temperature), soil properties (pH, N/P ratio, available phosphorus and soil water content), microbial carbon pools (microbial biomass C and microbial necromass C) and microbial community properties (bacterial diversity, fungal diversity, network PC1 and network PC2) were used to predict SOC. To examine the relationships between predictors and their correlations with SOC, we initially performed the Pearson correlation analysis. Due to the significant correlation between microbial necromass C and both fungal necromass C and bacterial necromass C, we selected only microbial necromass C as a predictor in the SOC models. We used multiple regression models to assess the effects of climate, soil properties, microbial carbon pools and microbial community properties on SOC [53]. All predictors and response variables were Z score standardized to interpret parameters on a comparable scale. Using the “MuMIn” package [54], we generated a set of models comprising all possible combinations of the initial predictors. The models were then ranked based on the Akaike information criterion (AIC) fitted with maximum likelihood in R. We selected all models with ΔAIC < 2 and used the model averaging approach to estimate parameters and associated *P*-values, using the function model.avg. We then calculated the relative effect of the parameter estimates for each of the predictors compared with the effect of all parameter estimates. This method allowed us to evaluate the identifiable relative importance of climate, soil properties, microbial carbon pools and microbial community properties in predicting SOC [53, 55]. In addition, to explore the importance of microbial carbon pools and community properties for predicting SOC, we built competing models without microbial carbon pools and community properties. Thus, four models were built (model #1 to #4). Model #1 included all climate and soil property predictors as well as microbial carbon pools and community properties. Model #2 included the predictors of model #1 except microbial community properties. Model #3 included the predictors of model #1 except microbial carbon pools. Finally, model #4 included only climate and soil property predictors excluding both microbial community properties and carbon pools. The best model among the four models was assessed by using the AIC value; that is, the lower AIC, the better of model.

Additionally, the structural equation modeling (SEM) was adopted to explore the pathways of microbial C pools and community properties in driving SOC. We first removed the effects of climate and soil properties on SOC by fitting a multiple regression model including those predictors and saved the residuals. Then we started the SEM procedure with the hypothetical relationships between microbial C pools, community properties and SOC residuals. In the SEM analysis, we compared the model-implied variance-covariance matrix against the observed variance-covariance matrix. Data were fitted to the models using the maximum-likelihood estimation method. Model fit statistics included the Chi-square (χ^2^), probability level (*P*), R^2^ (proportion of variance explained), and comparative fit index (CFI). The SEM analysis was conducted in the environment of Amos 20.0 (Amos Development Company, USA).

## Results

### Soil carbon pools and properties

**Fig. 1:**
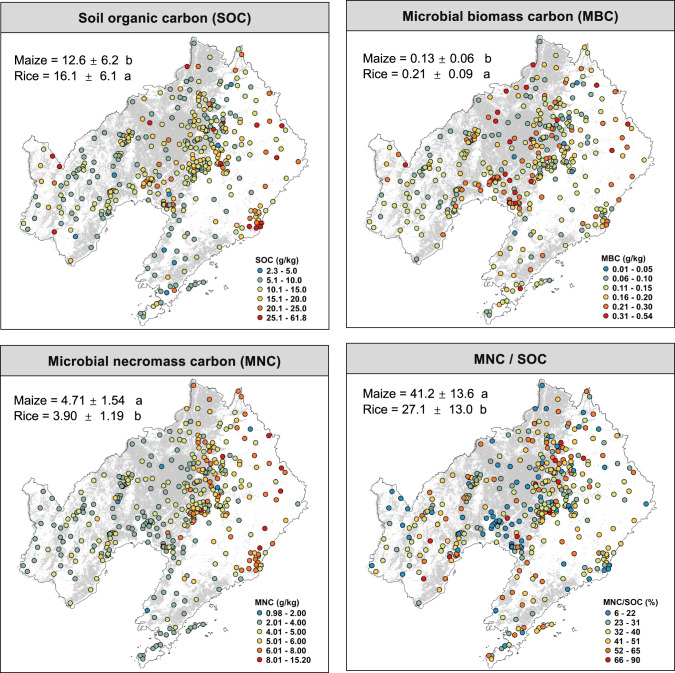
Geographical distribution of soil and microbial carbon pools. The four panels represent the soil organic carbon (SOC), microbial biomass carbon (MBC), microbial necromass carbon (MNC) and the ratio of necromass carbon to soil organic carbon (MNC/SOC). Mean and standard deviations for each variable are reported in the top left corner of each panel and means with the same lower case are not significant at *P* < 0.05 between maize and rice.

The SOC and microbial biomass C were significantly different between maize and rice (Fig. 1). On average, the SOC under rice (16.1 ± 6.1) was significantly higher than that under maize (12.6 ± 6.2), and the microbial biomass C showed a similar pattern (rice 0.21 ± 0.09 vs. maize 0.13 ± 0.06). Maize soil had greater microbial necromass C (4.71 ± 1.54), bacterial necromass C (1.22 ± 0.42) and fungal necromass C (3.49 ± 1.24) than rice soils (microbial necromass C, 3.90 ± 1.19; bacterial necromass C, 1.01 ± 0.53; fungal necromass C, 2.91 ± 0.89) (Supplementary Table S1). Moreover, microbial necromass C accounted for 41.2% for maize soils but only 27.1% for rice soils (Fig. 1). Besides, the soil water content, total nitrogen, and N/P ratio were higher in rice soils compare with  maize, while soil phosphorus and available phosphorus were higher in maize soils (Supplementary Table S1).

### Microbial diversity and network complexity

**Fig. 2:**
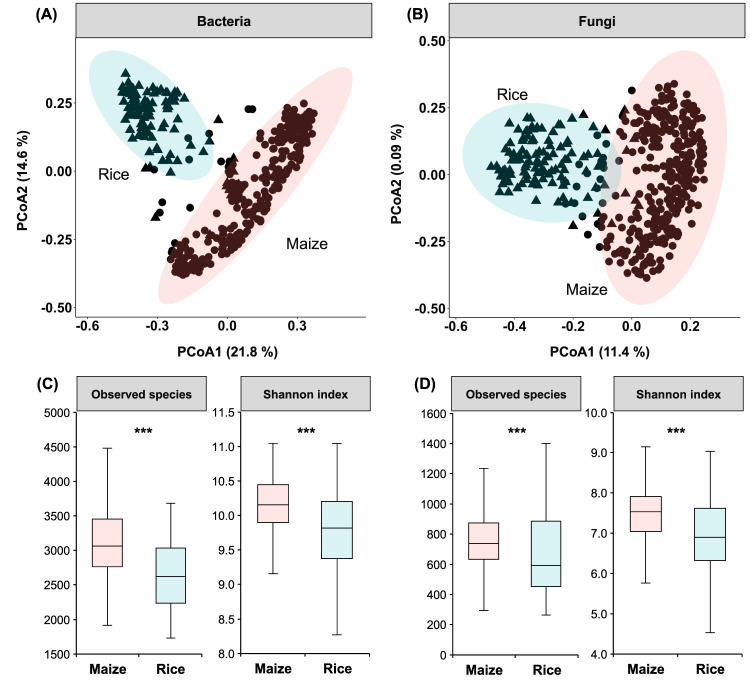
Soil microbial community composition and diversity. Principal coordinates analysis (PCoA) with Bray-Curtis distance showing that **A** bacterial and **B** fungal community composition of maize soils are significantly different from those of rice (PERMANOVA, *P* < 0.001). Ellipses cover 95% of the data for each crop. Alpha diversity indices (observed species and Shannon index) of **C** bacterial and **D** fungal communities in maize and rice. The asterisks indicate a significant difference between rice and maize soil at *P* < 0.001.

**Fig. 3:**
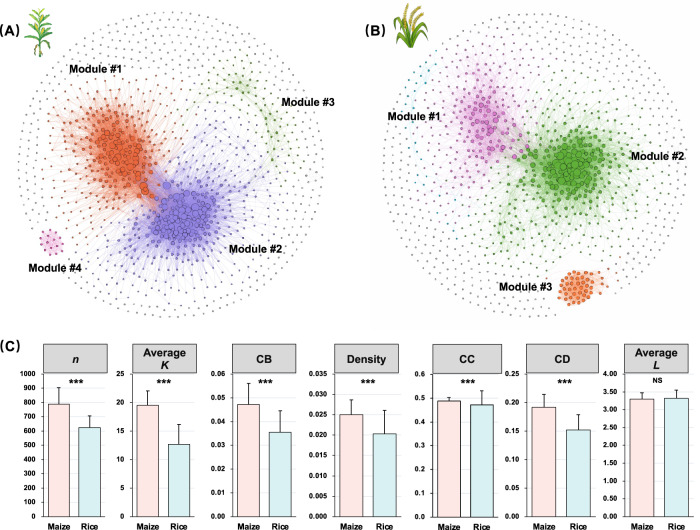
The microbial community network of soil bacteria and fungi. Network diagram with nodes colored according to the main modules for **A** maize and **B** rice. Nodes represent individual OTUs; edges represent significant Spearman correlations. **C** Average topological properties of microbial network for the studied sites. The network topological properties include node number (*n*), average connectivity (average *K*), centralization of betweenness (CB), density, clustering coefficient (CC), centralization of degree (CD) and average path length (average *L*). The asterisks indicate a significant difference between rice and maize soil. **P < 0.01; ***P < 0.001; NS not significant.

Principal coordinate analysis (PCoA) showed that maize and rice had clearly distinct bacterial and fungal community compositions (Fig. 2). Maize soils had greater alpha diversity than rice soils for both bacterial and fungal communities. To identify the potential species interactions in maize and rice, two co-occurrence networks were constructed (Fig. 3). The degrees (number of connections per node) within the two networks exhibited power-law distributions (Supplementary Fig. S2), which indicates a scale-free network structure and a non-random co-occurrence pattern. When we compared the network topological properties of the maize and rice soils, microbial co-occurrence patterns in the maize and rice soil were markedly different (Supplementary Table S2). In addition, the maize network differed from the rice network regarding the taxonomic composition of modules in the networks (Supplementary Fig. S3). In the maize network, modules #1 and #2 were dominated by Proteobacteria and Actinobacteria. Proteobacteria and Acidobacteria were predominant in module #3, whereas module #4 was dominated by fungal taxa (Auriculariales and Chaetothyriales). In the rice network, Proteobacteria and Chloroflexi were the two dominant taxa in modules #1 and #2; and Mortierellales was the most important taxa in module #3. Finally, the topological properties of the maize network were markedly different from those of the rice network (Fig. 3C). The PC1 and PC2 of microbial network properties accounting for 44.2% and 23.8% for maize, and 48.6% and 21.6% for rice (Supplementary Fig. S4) were used to denote the microbial network complexity index in SOC model building.

### Linking microbial community, biomass and necromass C to SOC

**Fig. 4:**
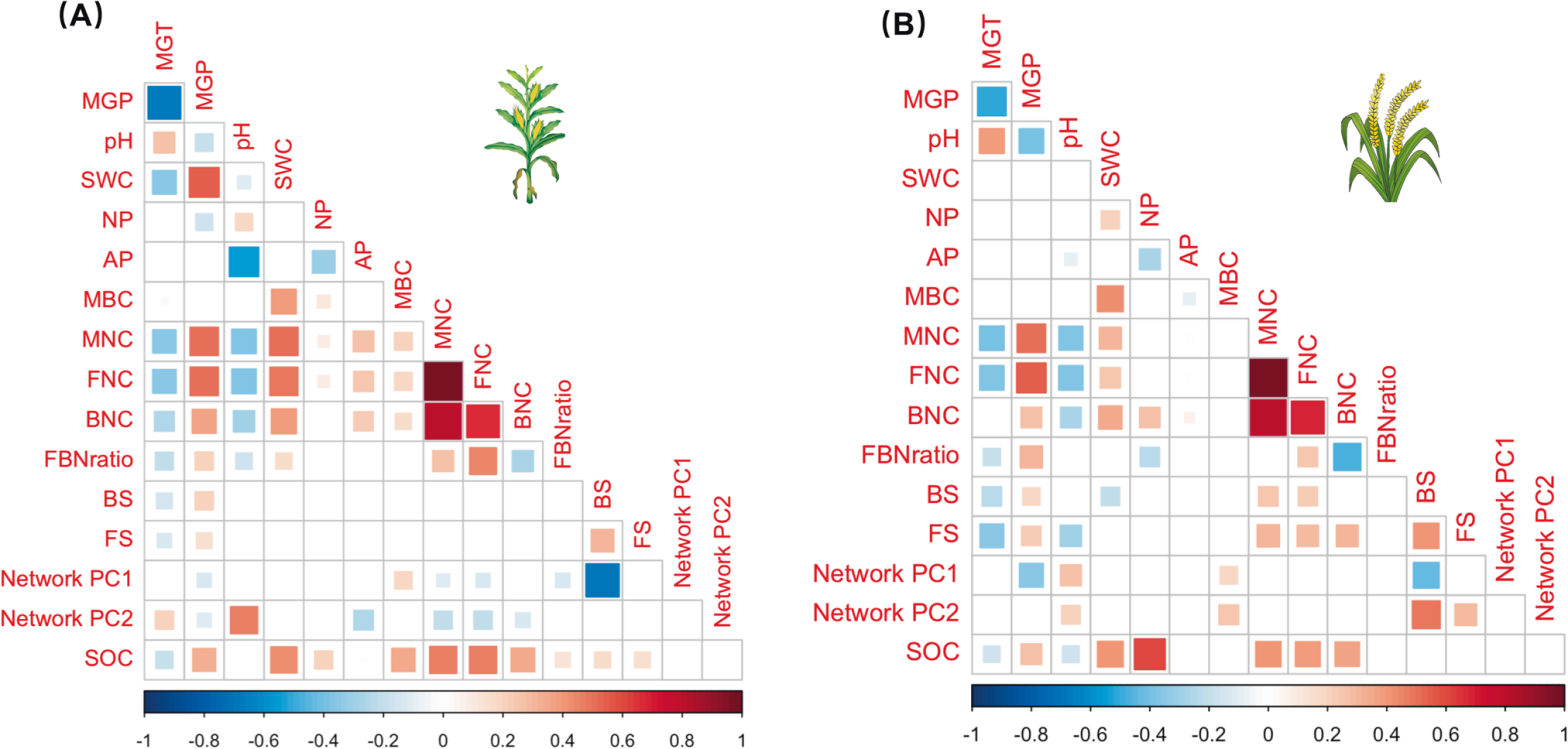
Investigate the correlation between soil organic carbon and its predictors. Heatmap of correlation between soil organic carbon and its predictors for **A** maize and **B** rice soil. MGP mean growing season precipitation, MGT mean growing season temperature, pH soil pH, SWC soil water content, NP soil nitrogen and phosphorus ratio, AP soil available phosphorus, MBC microbial biomass carbon, MNC microbial necromass carbon, FNC fungal necromass carbon, BNC bacterial necromass carbon, FBNratio the ratio of fungal necromass carbon to bacterial necromass carbon, BS bacterial Shannon index, FS fungal Shannon index, Network PC1 the first principal component of seven microbial topological properties, Network PC2 the second principal component of seven microbial topological properties, SOC soil organic carbon.

**Fig. 5:**
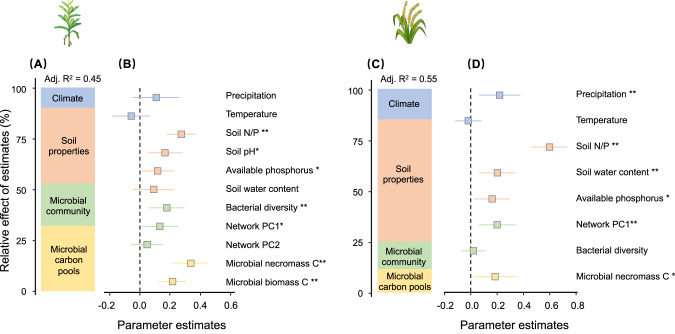
Relative effects of multiple predictors on soil organic carbon. The averaged parameter estimates (standardized regression coefficients) of the model predictors are shown with their associated 95% confidence intervals (**A** and **C**) along with the relative importance of each predictor, expressed as the percentage of explained variance (**B** and **D**) for maize and rice. The best model is selected based on the AICc (Supplementary Table S3). The relative effect of the predictors is calculated as the ratio between the parameter estimate of the predictor and the sum of all parameter estimates, and it is expressed as a percentage. The climate includes mean growing season precipitation (Precipitation) and mean growing season temperature (Temperature) at each location; soil properties include soil nitrogen and phosphorus ratio (soil N/P), soil pH, soil available phosphorus and soil water content; microbial community properties include bacterial diversity and network compelxity (Network PC1 and PC2); soil microbial carbon pools include microbial biomass and necromass carbon. *P < 0.05; **P < 0.01.

Microbial necromass C and its components (bacterial necromass C and fungal necromass C) were positively correlated with SOC in both maize and rice soils (Fig. 4). Further, microbial biomass C positively correlated with SOC maize but not for rice soils (Fig. 4). The best linear model for predicting SOC for both maize and rice included all predictors (model #1) and had both the lowest AIC and highest R^2^ (Fig. 5 and Supplementary Table S3). Removing either the microbial biomass C pools (model #2), microbial community properties (model #3), or both (model #4) from model #1 significantly reduced the predictive power (ΔAIC > 2, Supplementary Table S3). In the best model for maize, the microbial biomass C, necromass C, bacterial diversity, and network complexity (network PC1) were the significantly predictors of SOC (Fig. 5A). Microbial C pools (biomass and necromass) were responsible for 31.7% while community properties (diversity and complexity) were responsible for 23.9% of the explained variance in SOC for maize (R^2^ = 0.45). In the best model for rice, soil properties explained the largest proportion of SOC variation (57.7%), and the soil N/P ratio was the most important predictor of SOC. Together, microbial necromass C and microbial community parameters (network PC1 and bacterial diversity) explained a total of 25.7% of the variance in SOC in rice soil (Fig. 5B).

**Fig. 6:**
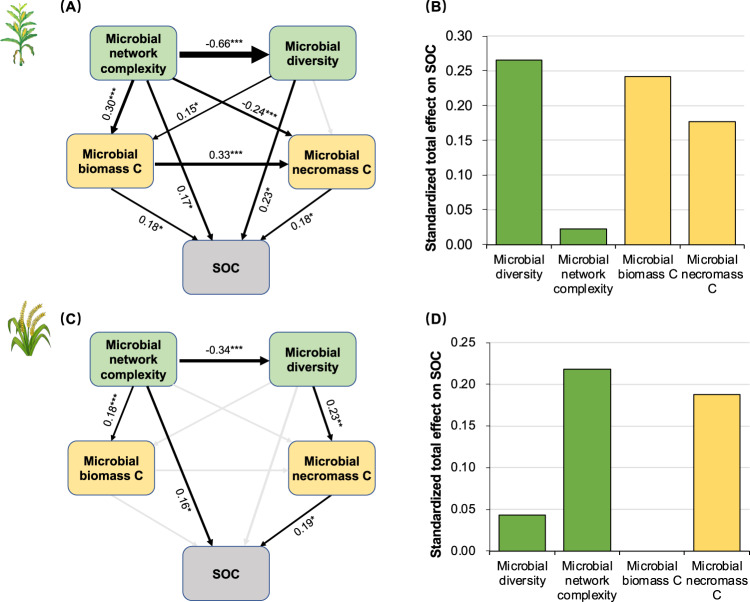
Structural equation modeling describing the roles of microbial community and C pools in predicting soil organic carbon. Before starting the modeling, the residual variance of soil organic carbon is calculated by accounting for climate and soil properties. Such residual variance represents 32% and 49% of the original SOC in the maize and rice soil, respectively. The linkages among microbial diversity, network complexity, biomass C and necromass C to SOC for **A** maize and **C** rice. Numbers adjacent to arrows are standardized path coefficients, analogous to relative regression weights, and indicative of the effect size of the relationship. Gray arrows indicate non-significant paths. Goodness-of-fit tests for maize: χ^2^ = 2.61, *P* = 0.106, GFI = 0.994, RMSEA = 0.070; Goodness-of-fit tests for rice: χ^2^ = 3.64, *P* = 0.602, GFI = 0.988, RMSEA = 0.000. *P < 0.05; **P < 0.01; ***P < 0.001. Standardized total effects from structural equation modeling for **B** maize and **D** rice.

Potential microbial-mediated mechanisms of SOC accrual in cropland systems were explored using SEM (Fig.6). The SEM for maize soil suggested that necromass C and biomass C may have direct and positive effects on SOC, while microbial network and diversity influenced SOC directly or indirectly through microbial biomass C and necromass C (Fig. 6A). For rice soil, the SEM suggested necromass C may positively influence SOC, while microbial biomass C does not (Fig. 6C). The microbial network parameters linked directly to SOC and indirectly via microbial diversity and necromass C in the rice SEM. When the standardized path coefficients of the microbial community and carbon pools were summed, the results suggest microbial diversity and microbial biomass C were the first and second most important microbial predictors for SOC in maize (Fig. 6B), while the microbial network properties and necromass C were the first and second most important microbial predictors for SOC in rice soil (Fig. 6D).

## Discussion

Soil organic carbon in cropland is highly dynamic, and increasing SOC could potentially promote crop production [56] as well as C sequestration [10, 57]. In comparison to natural ecosystems, cropland tends to have lower SOC because of the irrigation-associated erosion, chemical fertilizer application, and removal of crop residues [3, 5, 11]. Improved cropland management, such as cover cropping and no tillage, have the potential to increase both the quantity and quality of SOC. However, the underlying mechanisms responsible for the increase in SOC resulting from these management practices remain unclear, particularly with respect to the roles of soil microorganisms [2, 10, 58]. In this study, we conducted a large-scale soil survey in an important crop production region of China (Supplementary Fig. S1). The results demonstrate that considering microbial properties related to life and death (e.g., microbial diversity, network complexity, biomass and necromass) could better improve the predictions of cropland SOC. These findings highlight the importance of microbial community in regulating SOC formation and have potential implications for the C management in croplands.

### Contribution of necromass C to SOC in cropland

The importance of microbial biomass and necromass to SOC formation and prediction in natural ecosystems has been investigated [25, 59], yet the significance of these factors for cropland SOC remains unclear. This study is the first, to our knowledge, to show the regional-scale links among the microbial biomass C, necromass C, and SOC in cropland soils. The positive correlation between microbial necromass (assessed via amino sugars) and the content of SOC for both rice and maize systems (Fig. 4) supports our hypothesis that microbial necromass is an important contributor to soil organic matter [15, 22, 28]. However, the importance of microbial necromass C may depend upon the crop. The average SOC content in rice soil was higher than that in maize soil (Fig. 1), but the ratio of necromass C to SOC was the opposite (27.0% in rice vs. 41.2% in maize). This is likely because waterlogged rice soils experience oxygen limitation which slows microbial anabolism, allowing the refractory components of plant residues to accumulate and decreasing the formation of microbial necromass [33].

Additionally, fungal necromass C accounted for the majority of necromass C with an average of 74% (46–85%) in maize and 75% (33–88%) in rice soil (Supplementary Table S1). This suggests that fungi may be a more important source of SOC than bacteria in studied croplands. The higher accumulation of fungal necromass in soil could be due to its lower decomposition rate in soil and higher recalcitrant C components [20, 26, 60]. Moreover, because fungi could facilitate aggregate formation, the cell fragments of fungal necromass can have a higher chance of being physically protected by aggregate cohesion [61–63]. Taken together, these findings suggest that fungal necromass, rather than bacterial necromass, played a more important role in the accumulation of SOC in the studied cropland soil.

### Importance of microbial community properties to soil C pools

Linking microbial community diversity and composition to soil C storage has been a challenging task in soil ecology [14, 64, 65]. Our study provides novel evidence that microbial community properties may influence SOC accumulation through their effect on microbial biomass and necromass. Specifically, our results demonstrate significant correlations between microbial community complexity, diversity, and soil organic carbon. Maize soil had a higher microbial diversity and complexity along with microbial necromass C pool and necromass C to SOC ratio relative to rice soils (Fig. 1). This may be explained by the following two reasons. First, microbial diversity and community complexity influence microbial community carbon use efficiency [30]. Microbial diversity often positively relates to microbial carbon use efficiency (CUE), resulting in more microbial biomass production and necromass C retention in soil [30, 66, 67]. Indeed, a recent global analysis suggests high CUE allows more allocation to biomass and by-products, which leads to SOC accumulation [68]. Second, microbial death processes could be affected by microbial community dynamics and inter-species interactions [24]. For instance, competition for resources within a microbial community may lead to higher starvation and death, resulting in increased production of microbial necromass and subsequent SOC buildup [15, 24]. Overall, our study, along with previous studies, supports an overall life cycle view to describe the role of microorganisms in SOC dynamics, which includes the microbial community, growth, biomass, turnover and necromass [15, 29]. This view emphasizes that cropland SOC production would be promoted not only by increasing microbial-derived C production, but also by fostering microbial community properties that facilitate higher carbon use efficiency and favorable inter-species interactions.

### Prediction of SOC by integrating the microbial community

As hypothesized, integrating microbial parameters improved the model’s accuracy in predicting SOC. Specifically, regression models that exclude both microbial biomass and necromass C had lower prediction power (Supplementary Table S3). This finding aligns with other studies that have demonstrated the value of including microbial properties to enhance SOC predictions [25, 68]. For example, including microbial necromass improved the performance of both first-order kinetic and Michaelis-Menten model [17]. Connecting microbial community properties with SOC has also been attempted in previous studies. For instance, recent work suggests soil bacterial communities to be utilized as bioindicators of SOC [69]. Our results go beyond these previous works by simultaneously integrating both microbial community properties and microbial carbon pools to predict SOC in croplands. But it should be noted that the relative importance of microbial carbon pools and community properties on SOC is difference between maize and rice soils. To better integrate microbes in SOC models, we propose a framework that considers “microbial living biomass”, “necromass” and “microbial community” as three crucial predictors (similar to our SEM, Fig. 6) and change the parameters when predicting SOC for different crop types (e.g., maize vs. rice). Moreover, our results also indicate that the relative abundance of microbial composition is a poor indicator of SOC in the studied soils (data not shown). Understanding the environmental conditions and ecological processes that govern microbial community properties and C pools will be critical to enhancing SOC sequestration.

### The importance of soil properties for SOC in cropland

Our results suggest that soil properties also play an important role in determining SOC concentration in cropland soils. In particular, we found that the soil nitrogen and phosphorus ratio and available phosphorus were positively correlated with SOC (Fig. 5). This finding is supported by past studies that nutrient sufficiency can facilitate SOC increases in cropland soils [70, 71]. One possible reason is that increasing nitrogen and phosphorus can increase crop biomass, allowing more plant-derived C to enter the soil for accumulation [72]. Another possibility is that changes in the N and P supply can affect living microbial biomass, microbial community composition and necromass production [71, 73]. Further research should work to separate the direct and indirect roles of soil nutrient conditions on C storage in croplands and estimate the relative contribution of each pathway.

## Conclusion

In summary, it remains a challenge to manipulate microbial community composition and function for maximum organic C storage in cropland soils. However, the present study provides novel insights into the correlation between microbial necromass and SOC in cropland on a regional scale. Specifically, we found that fungal necromass had a greater contribution than bacterial necromass to the accumulation of SOC. Additionally, our results suggest that microbial diversity, community complexity, and microbial living biomass C either directly or indirectly influence microbial necromass C and ultimately impact on SOC. This research contributes to our understanding of how microbial life and death impact SOC and suggests that management practices targeting these microbial parameters may enhance cropland SOC.

### Supplementary information

Supplementary Information

## Data Availability

Microbial amplicon sequencing data were deposited to the Science Data Bank and were retrievable using the link: 10.57760/sciencedb.06368.
