# Supplementary material for: Integrating microbial community properties, biomass and necromass to predict cropland soil organic carbon
Source: ISME Commun. 2023 Aug 23;3:86. doi: 10.1038/s43705-023-00300-1 (PMC10447565; doi:10.1038/s43705-023-00300-1)
Supplement: Supplementary file 1 — Supplementary Information [file 43705_2023_300_MOESM1_ESM.docx]

Supplementary information for

Integrating microbial community properties, biomass and necromass to predict cropland soil organic carbon

Chao Wang ^1,2 *^, Xu Wang ^1^, Yang Zhang ^1^, Ember Morrissey ^3^, Yue Liu ^1^, Lifei Sun ^1^, Lingrui Qu ^1^, Changpeng Sang ^1^, Hong Zhang ^1^, Guochen Li ^1, *^, Lili Zhang ^1^, Yunting Fang ^1^

^1^ CAS Key Laboratory of Forest Ecology and Management, Institute of Applied Ecology, Chinese Academy of Sciences, Shenyang, 110016, China

^2^ Key Laboratory of Terrestrial Ecosystem Carbon Neutrality, Liaoning Province, Shenyang, 110016, China

^3^ Division of Plant and Soil Sciences, West Virginia University, Morgantown, 26506, USA

***Corresponding author**

Chao Wang & Guochen Li

Institute of Applied Ecology, Chinese Academy of Sciences

No. 72 Wenhua Road

Shenyang, Liaoning

110016, China

Telephone: +86-24-83970570

Email: cwang@iae.ac.cn (C.W.) & ligc@iae.ac.cn (G.L)

**Contents of this file**

Tables S1 to S3

Fig. S1 to S4

**Table S1. Basic information of the sampling sites (mean ± SD).**

| **Variables** | **Maize** | **Rice** |
| --- | --- | --- |
| Latitude (Nº) | 38.87-43.37 | 39.21-43.00 |
| Longitude (Eº) | 119.0-125.4 | 118.9-125.5 |
| Mean growing season precipitation (mm) | 259-753 | 379.7-759.3 |
| Mean growing season Temperature (℃) | 5.5-10.4 | 6.7-10.1 |
| Soil water content (%) | 14.8 ± 6.37 **b** | 35.3 ± 8.82 **a** |
| Soil pH | 6.26 ± 0.58 **a** | 6.39 ± 0.50 **a** |
| Soil total nitrogen (g kg^-1^) | 1.14 ± 0.46 **b** | 1.38 ± 0.45 **a** |
| Soil total phosphorus (g kg^-1^) | 0.74 ± 0.34 **a** | 0.65 ± 0.20 **b** |
| Soil N/P ratio | 1.69 ± 0.61 **b** | 2.23 ± 0.73 **a** |
| Soil avaiable phosphorus (g kg^-1^) | 0.038 ± 0.028 **a** | 0.020 ± 0.015 **b** |
| Soil organic carbon(g kg^-1^) | 12.6 ± 6.2 **b** | 16.1 ± 6.1 **a** |
| Microbial biomass carbon (g kg^-1^) | 0.13 ± 0.06 **b** | 0.21 ± 0.09 **a** |
| Microbial necromass carbon (g kg^-1^) | 4.71 ± 1.54 **a** | 3.90 ± 1.19 **b** |
| Bacterial necromass carbon (g kg^-1^) | 1.22 ± 0.42 **a** | 1.01 ± 0.53 **b** |
| Fungal necromass carbon (g kg^-1^) | 3.49 ± 1.24 **a** | 2.91 ± 0.89 **b** |
| Fungal necromass/ bacterial necromass C | 2.93 ± 0.74 **b** | 3.19 ± 0.78 **a** |
| Fungal necromass / Microbial necromass C | 0.74 ± 0.06 **b** | 0.75 ± 0.06 **a** |

Different letters indicate significant difference between mazie and rice for a given variable.

**Table S2. Key toplogical properties of microbial community networks of maize and rice.**

|  | **Netork topoligical properties** | **Maize** | **Rice** |
| --- | --- | --- | --- |
| Empirical network | Node number | 1162 | 1108 |
|  | Edge number | 12160 | 8555 |
|  | Average connectivity | 20.93 | 15.44 |
|  | Centralization of betweenness | 0.04 | 0.02 |
|  | Density | 0.02 | 0.01 |
|  | Clustering coefficient | 0.47 | 0.46 |
|  | Centralization of degree | 0.15 | 0.11 |
|  | Average path length | 3.39 | 3.45 |
|  | Modularity | 0.47 | 0.52 |
| Random  network | Clustering coefficient ± SD | 0.18 ± 0.009 | 0.13 ± 0.01 |
|  | Average path length ± SD | 2.91 ± 0.01 | 3.07 ± 0.02 |

**Table S3. Results of model selection.** Model selection based AICc of the best set of predictors on explaining soil organic carbon. Model 1 includes all predictors. Model 2 includes the predictors of model 1 expect microbial community properties. Model 3 includes the predictors of model 1 expect microbial carbon pools. Model 4 includes the predictors of model 1 except for both microbial community and carbon pools. The R^2^, adjusted R^2^, AICc, and ΔAICc of each best model are shown for maize and rice soil. The climate includes mean growing season precipitation (Precipitation) and temperature (Temperature) at each location; soil properties include soil nitrogen and phosphorus ratio (N/P), soil pH, soil available phosphorus and soil water content; microbial communities include bacterial diversity (bacterial Shannon index and fungal Shannon index) and network complexity (Network PC1 and Network PC2); soil microbial carbon pools include microbial biomass and necromass carbon. The colors indicate the predictors were selected. Dashed lines indicate the predictors are not considered in building each model.

| **Models** | | **Maize** | | | | **Rice** | | | |
| --- | --- | --- | --- | --- | --- | --- | --- | --- | --- |
|  |  | **Model**  **1** | **Model**  **2** | **Model 3** | **Model 4** | **Model 1** | **Model 2** | **Model 3** | **Model 4** |
| Model parameters | R^2^ | 0.46 | 0.44 | 0.36 | 0.33 | 0.58 | 0.55 | 0.56 | 0.52 |
|  | Adj R^2^ | 0.45 | 0.43 | 0.35 | 0.32 | 0.55 | 0.52 | 0.54 | 0.50 |
|  | AICc | 749.22 | 757.47 | 801.01 | 810.62 | 242.29 | 248.49 | 245.73 | 251.64 |
|  | ΔAICc | 0.00 | 8.25 | 51.79 | 61.40 | 0.00 | 6.20 | 3.44 | 9.35 |
| Climate | Precipitation |  |  |  |  |  |  |  |  |
|  | Temperature |  |  |  |  |  |  |  |  |
| Soil  properties | Soil N/P |  |  |  |  |  |  |  |  |
|  | Soil available phosphorus |  |  |  |  |  |  |  |  |
|  | Soil pH |  |  |  |  |  |  |  |  |
|  | Soil water  content |  |  |  |  |  |  |  |  |
| Microbial community | Bacterial  diversity |  |  |  |  |  |  |  |  |
|  | Fungal  diversity |  |  |  |  |  |  |  |  |
|  | Network PC1 |  |  |  |  |  |  |  |  |
|  | Network PC2 |  |  |  |  |  |  |  |  |
| Microbial carbon pools | Microbial  biomass C |  |  |  |  |  |  |  |  |
|  | Microbial  necromass C |  |  |  |  |  |  |  |  |

**Fig. S1. Study region and sampling sites. (A)** The study region (Liaoning province) locates in northeast China. The green background indicates cropland area in China. (**B**) Spatial distribution of sampling sites, including maize (n = 349) and rice (n = 119).

**Fig. S2. Degree distribution of (A) maize and (B) rice microbail community networks.**

**Fig. S3. Phylum-level relative abundance of the dominant modules for (A) maize and (B) rice microbial community networks.**

**Fig. S4. Principal components of microbial network properties for (A) maize and (B) rice.** Microbial network topological properties include node number, degree, betweenness centrality, graph density (Density), clustering coefficient, centralization of degree, and average path length. The first and second components of this principal analysis were used to denote the network complexity.
